# Application of neural oscillators to study the effects of walking speed on rhythmic activations at the ankle

**DOI:** 10.1186/1742-4682-10-9

**Published:** 2013-02-13

**Authors:** Sook-Yee Chong, Heiko Wagner, Arne Wulf

**Affiliations:** 1 Department of Motion Science, University of Muenster, Horstmarer Landweg 62b, Muenster, 48149, Germany

**Keywords:** Locomotion, Walking speed, Silly walks, EMG, Spinal pattern generator, Neural network, Sensory afferents, Motor control

## Abstract

**Background:**

Spinal pattern generators (SPG) are neural networks in the spinal cord that do not require a central input from the brain to generate a motor output. We wanted to determine whether SPG can adapt to the changing motor demands from walking at different speeds, and performing silly walks.

**Methods:**

An SPG model consisting of an oscillator made up of two neurons was utilised in this study; one neuron activates the soleus and the other activates the tibialis anterior. The outputs of the SPG model therefore represent the electromyographic measurements from each muscle. Seven healthy subjects were requested to perform silly walks, normal walking at self-selected speed (4.8 ± 0.5 km/h), 3.5 km/h, 4.0 km/h and 4.5 km/h on a treadmill. Loading and hip angles were used as inputs into the model.

**Results:**

No significant differences in the model parameters were found between normal walking at self-selected speed and other walking speeds. Only the adaptation time constant for the ankle flexor during silly walks was significantly different from the other normal walking trials.

**Conclusion:**

We showed that SPG in the spinal cord can interpret and respond accordingly to velocity-dependent afferent information. Changes in walking speed do not require a different motor control mechanism provided there is no disruption to the alternating muscular activations generated at the ankle.

## Background

Afferents interact continuously with different parts of the nervous system so as to enable a smooth and efficient gait. As the human nervous system should coordinate efficiently, responding and adapting to the immediate environment, it is important that the plethora of signals coming from the central, sensory and peripheral systems be selected and modulated, so that the motor output fulfils the demands of the locomotor task.

Different control networks at different levels of the nervous system contribute to human motor control. The lowest level of neural control, which is responsible for generating the basic patterns of locomotion, is believed to come from spinal pattern generators (SPG) located in the spinal cord [1-3]. Brown [4] showed that decerebrate cats can produce locomotor-like electromyographic (EMG) patterns while walking on a treadmill. Similar results were also obtained from other vertebrates and invertebrates [1,5]. A study by Maegele et al. in 2002 [6] showed that clinically incompletely and completely spinal cord-injured patients can activate lower limb muscles after treadmill therapy. While these studies successfully showed the ability of the SPG to produce a motor output with no interference from the brain, they also demonstrated that the interactions between SPG and sensory inputs are important in generating a dynamic movement [7]. Taga [8] had shown that a real-time dynamic interaction between the neural and mechanical system, together with sensory information from the environment, could influence the motor output of the lower limbs.

Walking at a slower or faster pace creates different motor demands on the neural system. A number of gait components such as stance and swing phase intervals and muscle activations change with increasing speed. However, in healthy humans, it is not known whether these changes result from sensory cues to the neural network in the spinal cord, since higher commands from the brain can intervene. The aim of our study was to determine whether neural networks in the spinal cord can adapt to changing sensory afferents, and directly influence muscular activity to meet the motor demands of walking at different speeds. We therefore studied the response of the SPG model in situations where gait components in a gait cycle will be different from normal walking: change in walking speed and in performing “silly walks”. Our study used an SPG model that is triggered only by sensory afferents with no interference from a cortical signal.

## Methodology

Seven healthy male subjects (28.0 ± 4.4 years, 1.8 ± 0.1 m, 76.4 ± 9.5 kg) volunteered to participate in this study. They were thoroughly informed of the procedures and gave their consent. Each subject was requested to walk at his normal self-selected speed (4.8 ± 0.5 km/h), at 3.5 km/h, 4.0 km/h and 4.5 km/h on a treadmill (Kinetics s3, Kettler, Germany). In addition, they were asked to perform movements unlike their normal walking, i.e. “silly walks”, at a speed of their own choice (3.8 ± 0.4 km/h). Data from six consecutive strides were collected during steady-state walking. Three trials were recorded for each subject for each walking speed and silly walks, i.e. a total of 210 trials (for both right and left limbs). Trials were ignored if there were missing data in any one stride. Therefore, a total of only 176 trials was analysed in this study.

Vertical force data were collected at 200Hz and calculated from in-shoe pressure sensors (Gesellschaft für Biomechanik Münster, Germany) as a summation of the pressure acting on the entire area of the insole. Hip angles were acquired from an Oqus 3D motion analysis system (Qualisys, Sweden) at 100 Hz. This system used six infra-red cameras, which tracked a total of fifteen retro-reflective markers attached to the following body landmarks: lateral and medial knee, and four tracking markers on the thigh of each leg, sacrum, and left and right anterior superior iliac spine. Segment definitions and kinematic data were processed using Visual3D (C-Motion Inc, Maryland, USA). Muscle activation from the *m. soleus* (SOL) and *m. tibialis anterior* (TA) were captured using bipolar surface electrodes (5–700 Hz, Biovision, Wehrheim, Germany) at 2000 Hz. The SOL and TA muscles were chosen because they are the principal monoarticular plantarflexor and dorsiflexor muscles, respectively. Electrodes were placed according to recommendations by Hermens et al., 1999 [9]. The electromyographic (EMG) signals were centred, rectified and filtered using a fifth-order low-pass Butterworth filter with a cut-off frequency of 40Hz.

**Figure 1:**
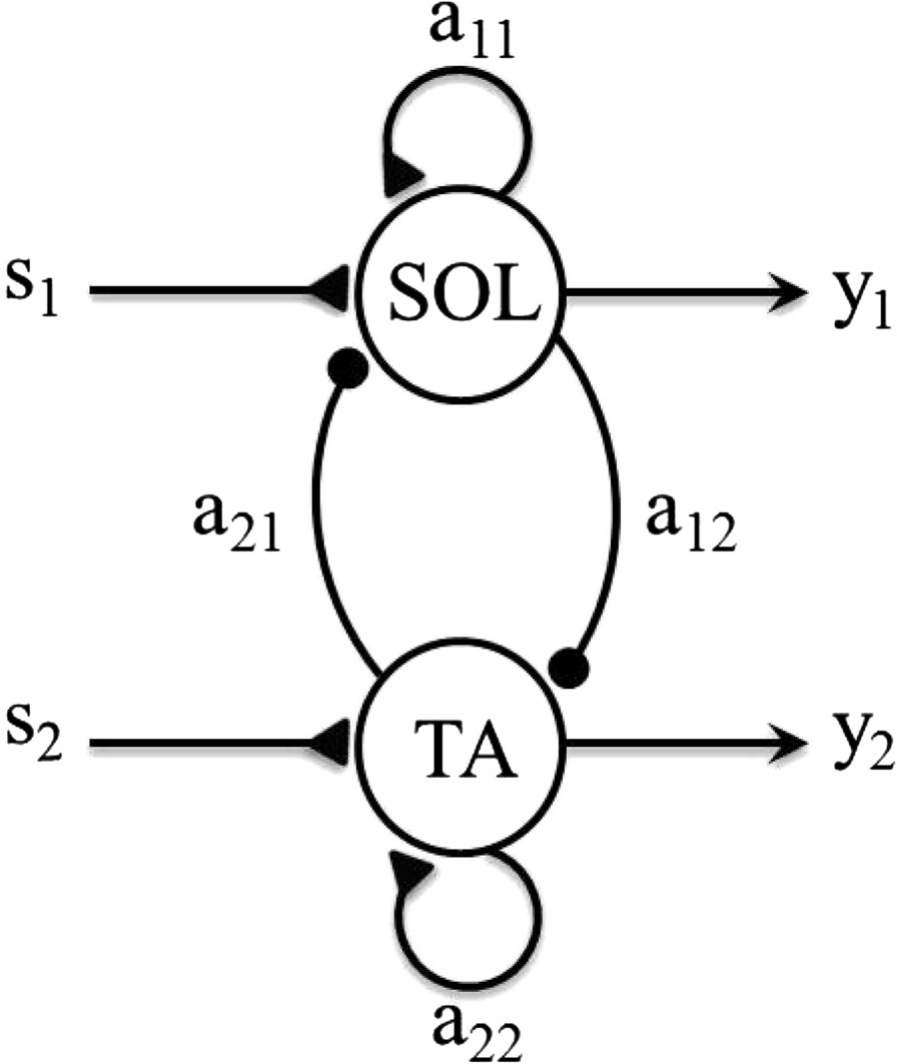
**SPG model consisting of two neurons.** Dark triangles represent excitatory connections, dark spheres represent inhibitory connections. Note that subscript 1 refers to SOL and subscript 2 to TA.

A simple Matsuoka oscillator [10,11] consisting of two neurons was used (Figure 1); one neuron activated the SOL and the other activated the TA. Thus, the outputs from the oscillator represented the corresponding activation of each muscle. The neurons were mutually inhibited, i.e. when one neuron was activated, the other was suppressed.

The oscillator was governed by the following equations (adapted from Matsuoka, 1985 [10]):

(1) $${\dot{x}}_{i}+x_{i}=\sum_{i}^{j}a_{\mathrm{ij}}y_{j}+s_{i}-b_{i}f$$

(2) $$T_{i}{\dot{f}}_{i}+f_{i}=y_{i}$$

(3) $$y_{i}=\max\left(0,x_{i}\right)$$

where *f* is the adaptation in the neuron, and *T* and *b* are the parameters that determine the time course of the adaptation. When *b* = 0 there is no adaptation and the output will increase and then remain at a constant value (Refer to Figure 1 in Matsuoka [10]). *x* is the inner state of the neuron, *y* is the generated output of the neuron, *s* is the input signal, and *a* is the strength of the connection between the two neurons; *a*_*ij*_ <0 for *i ≠ j* (mutual inhibition) and >0 for *i = j* (self-excitation). We assume a symmetrical arrangement of neurons, i.e. *a*_*ij*_ *= a*_*ji*_*, a*_*ii*_ *= a*_*jj*_*.*

The model is triggered by both the magnitude and the change in magnitude of loading and hip angles. Vertical force calculated from the insoles was first normalised to the subject’s weight. Normalised force *F* and hip flexion/extension angles *HA* of the ipsilateral limb (in radians) were used to determine the signal input *s*_*i*_ in equation (1):

(4) $$s_{i}=m_{i}.p+n_{i}.\dot{p}+w_{i}.q+v_{i}.\dot{q}$$

with

(5) $$\dot{p}=r_{1}\left(F-p\right)$$

(6) $$\dot{q}=r_{2}\left(\mathrm{HA}-q\right)$$

where *m, n, w and v* represent the weights of each excitation *p*,

$$\dot{p}$$,

*q*,

$$\dot{q}$$

respectively.

The parameters *a, b, m, n, r, T, v, w* from the above equations determined the pattern and frequency of the output. A nonlinear least-squares fitting algorithm was used to determine a set of parameters that would fit the output to experimental data i.e. the output produced by the neuron representing the SOL would be fitted to measured EMG data of the SOL. This was done simultaneously for the TA. Initial values for each neuron were taken from the first value of the measured EMG data so as to solve the differential equations numerically. The fitting algorithm terminated once the relative deviation between two iterations fell below 0.001. A correlation coefficient *R* between the model output and experimental EMG data was calculated in each trial.

The following gait components were analysed; Maximum normalised force and maximum range of hip flexion-extension angles were calculated for each stride. Stance and swing phases determined from force profiles of each stride were also calculated. For these gait components, analysis of variance (ANOVA) and Tukey’s post-hoc test were performed to determine the significant differences between all the different walking types. In analysing the rectified EMG signals for different speeds, we adopted the method by Murray et al., 1984 [12]. Cumulative numerical integration (IEMG) for each EMG signal in each stride was calculated for all speeds. The maximum of the mean IEMG values was designated as 1.00, regardless of speed. The other mean values were normalised with respect to this maximum value [12]. To determine significant differences in the model parameters (p < 0.05), multivariate analysis of variance (MANOVA) along with analysis of variance (ANOVA) and Tukey’s post-hoc test were performed.

## Results

**Figure 2:**
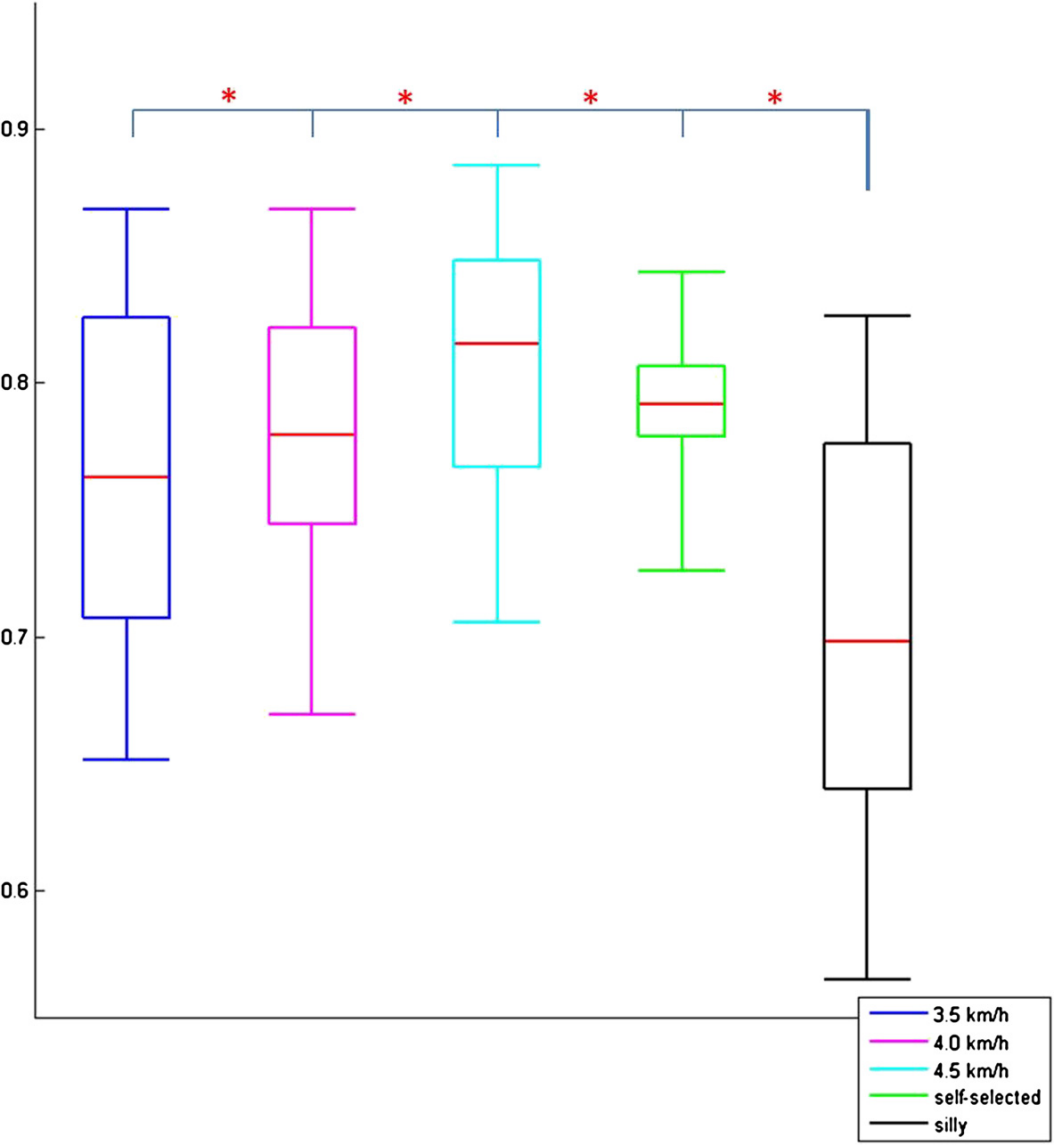
**Correlation coefficients (*R*) at different speeds and silly walks.** The tops and bottoms of the boxes are the 25th and 75th percentiles of *R* respectively. Red lines indicate the median values. (*) denotes significant difference.

**Figure 3:**
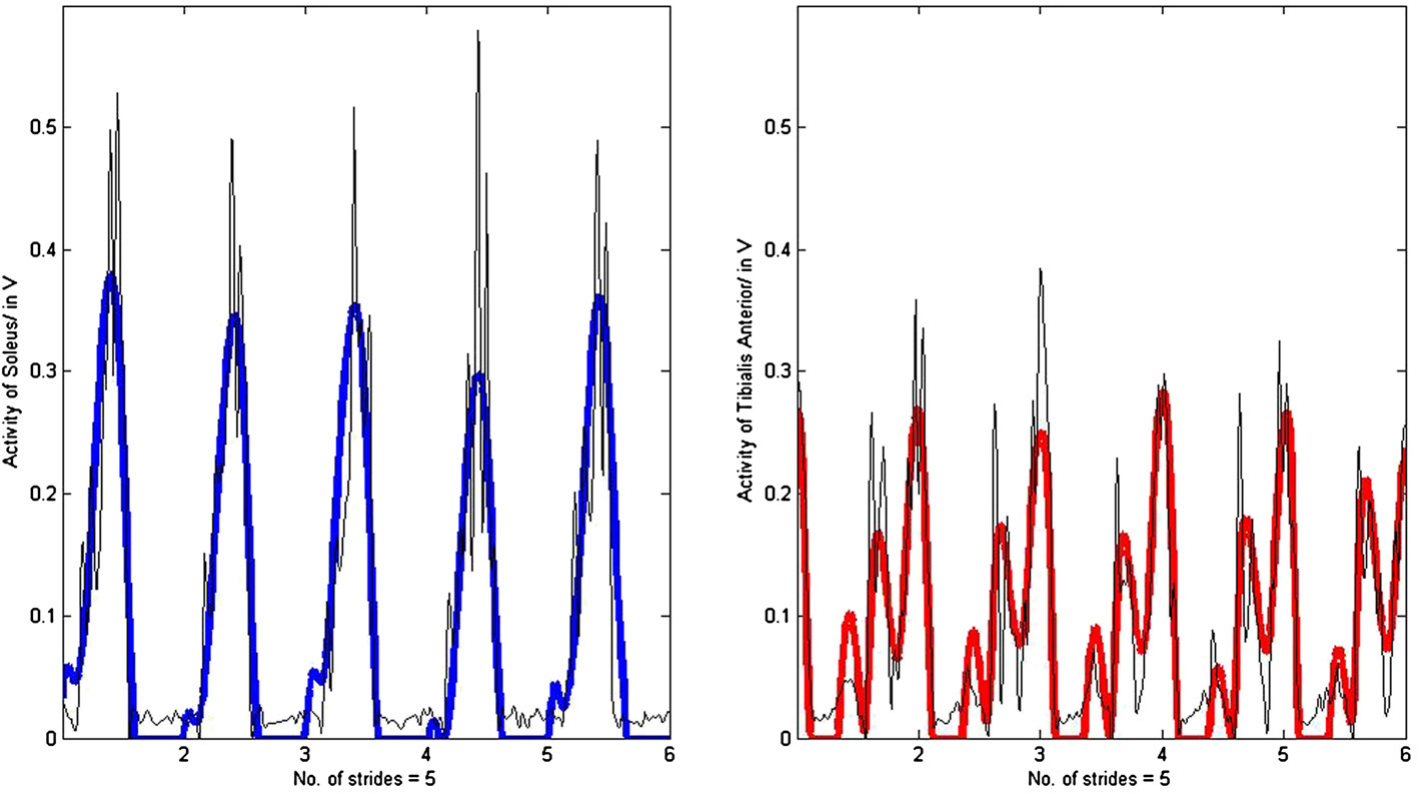
**R**_**average(ave)**_ **= 0.88.** Muscle activation of the soleus (R_SOL_ = 0.90) and the tibialis anterior (R_TA_ = 0.87) of subject #1 walking at 4.5km/h with insole forces and hip angles as inputs (bold lines represent the output from the SPG model and thin lines the experimental EMG data).

**Figure 4:**
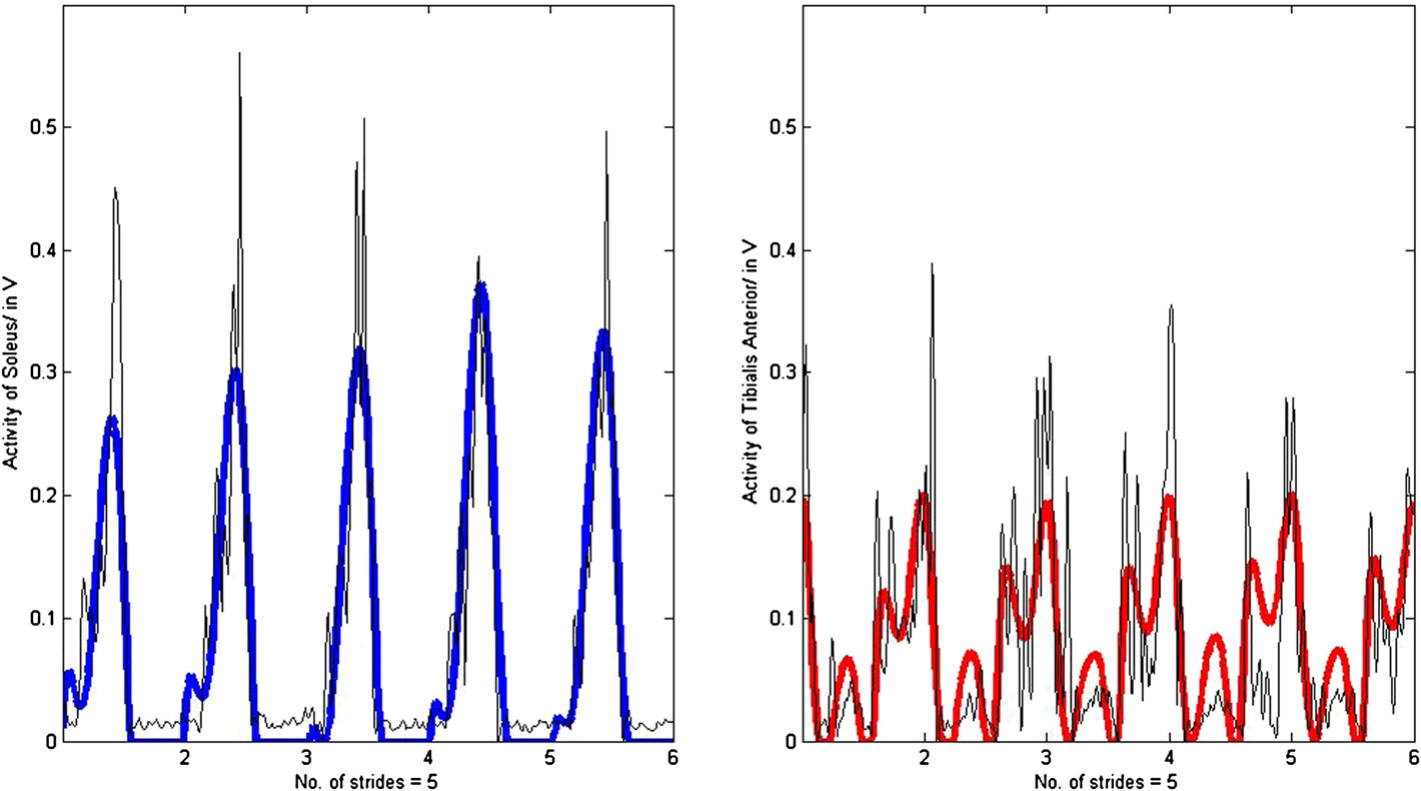
**R**_**ave**_ **= 0.80.** Muscle activation of the soleus (R_SOL_ = 0.90) and the tibialis anterior (R_TA_ = 0.71) of subject #1 walking at 4.0km/h with insole forces and hip angles as inputs (bold lines represent the output from the SPG model and thin lines the experimental EMG data).

**Figure 5:**
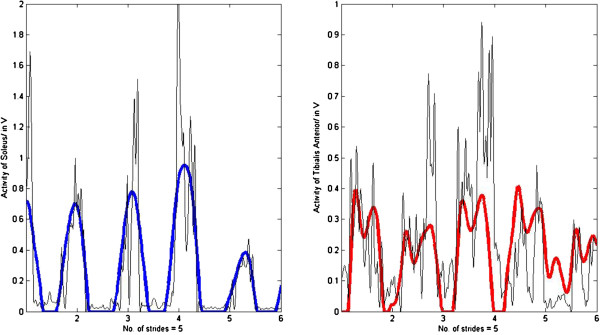
**R**_**ave**_ **= 0.63.** Muscle activation of the soleus (R_SOL_ = 0.79) and the tibialis anterior (R_TA_ = 0.46) of subject #1 performing silly walks with insole forces and hip angles as inputs (bold lines represent the output from the SPG model and thin lines the experimental EMG data).

No significant differences in *R* were found between normal walking at self-selected speeds and walking at other speeds. However, *R* calculated for silly walks (mean correlation R_mean_ = 0.70 ± 0.08) was significantly lower than for the other walking types (Figure 2). The quality of the fitting for silly walks is therefore not as good as for the other walking types (examples of three trials of one subject are presented in Figures 3, 4, 5). It was found that the output became oscillatory only after the first stride, so the results in Figures 3, 4, 5 are only from stride two onwards. It is also possible that the sensory inputs used in the model were insufficient to account for the muscular activations measured. Here, it is unknown whether additional sensory inputs or a cortical signal would give a better correlation.

Significant differences were found in the gait components calculated (Table 1). As expected, an increase in walking speed is accompanied by a decrease in the relative stance phase duration, an increase in the relative swing phase duration, an increased range of hip flexion-extension angles, and increased peak activation values of the SOL and TA [12]. Since loading and hip angles were significantly different, this meant that inputs to the SPG model differed significantly for all walking types.

**Table 1:** Mean and standard deviation (std) of gait components at different speeds and during silly walks

	** 3.5 km/h**		** 4.0 km/h**		** 4.5 km/h**		** Self-selected 4.8 ± 0.5 km/h**		** Silly walks 3.8 ± 0.4 km/h**		**p**
	**Mean**	**std**	**Mean**	**std**	**Mean**	**std**	**Mean**	**std**	**Mean**	**std**	
Stance (%)	66.60	±4.85	65.59	±4.34	65.22	±4.48	64.99	±1.58	63.33	±7.34	p < 0.05
Swing (%)	32.97	±3.01	34.05	±2.70	34.38	±2.79	35.01	±1.58	36.67	±7.34	p < 0.05
Hip flexion-extension range (rad)	0.67	±0.21	0.73	±0.22	0.76	±0.26	0.77	±0.07	0.84	±0.32	p < 0.05
Max F	1.11	±0.19	1.17	±0.22	1.23	±0.25	1.26	±0.20	1.25	±0.29	p < 0.05
IEMG_SOL	0.47	±0.26	0.51	±0.27	0.53	±0.31	0.53	±0.20	0.69	±0.20	p < 0.05
IEMG_TA	0.42	±0.26	0.47	±0.28	0.51	±0.32	0.53	±0.21	0.65	±0.21	p < 0.05

**Figure 6:**
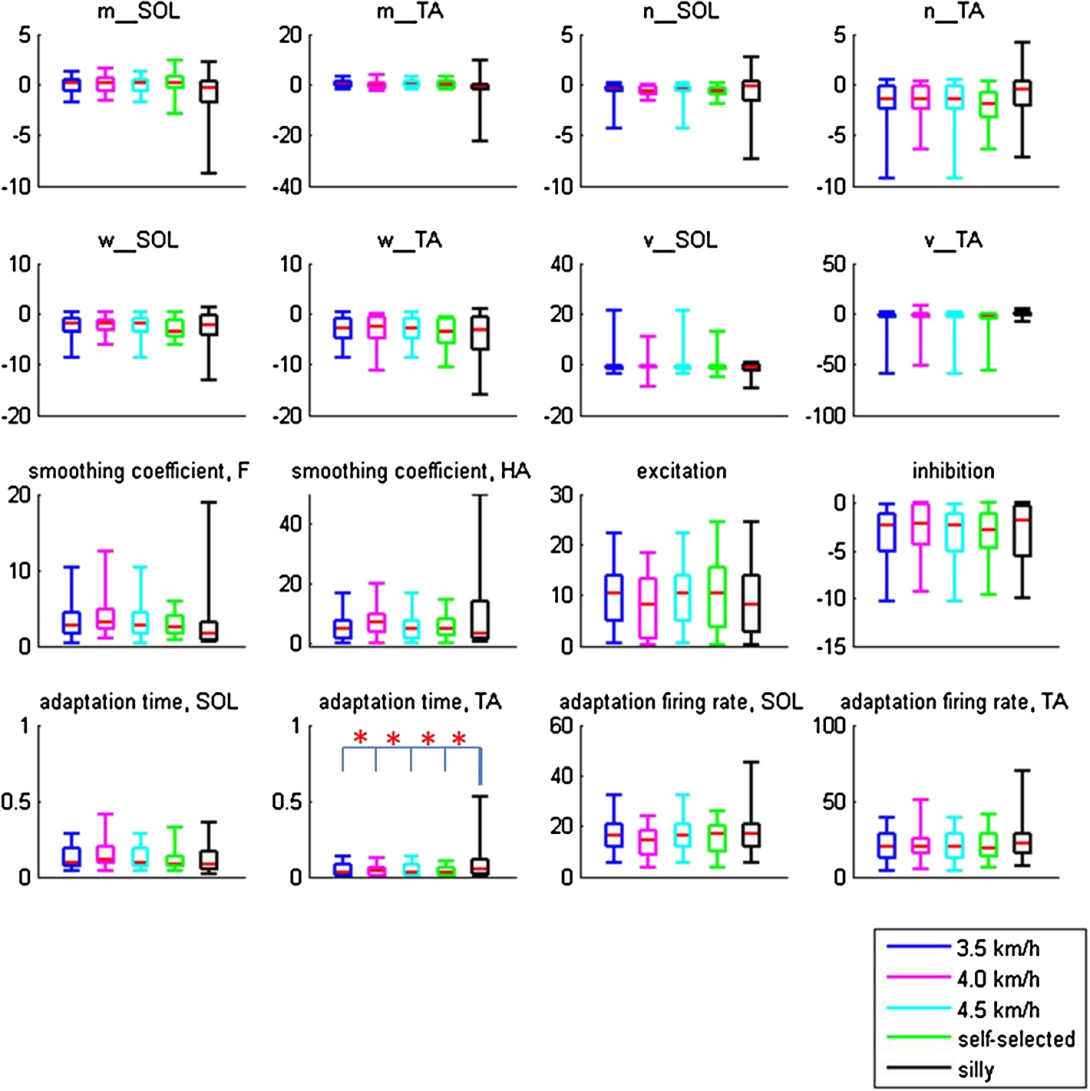
**Values of all parameters at different speeds and silly walks.** The tops and bottoms of the boxes are the 25th and 75th percentiles of the parameters respectively. Red lines indicate the median values. Parameters *m* and *n* are weights related to the normalised force *F*, while parameters *w* and *v* are weights related to hip angles *HA* (refer to equations 4–6). SOL: soleus, TA: tibialis anterior. (*) denotes significant difference.

MANOVA revealed significant differences between the model parameters. To continue with the analysis, ANOVA followed by Tukey’s post-hoc test revealed no significant differences between the model parameters for normal walking at self-selected speeds and other speeds (Figure 6). Only *T*_*2*_, the constant describing the time lag of the adaptation effect in the TA, showed significant differences between the silly walks and the normal walking trials.

## Discussion

The results of this study showed that neural networks in the spinal cord can activate muscles at the ankle to generate stepping motion during steady-state walking. In humans, it is difficult to determine whether the elevated EMG patterns during walking result from supraspinal control, activations from sensory inputs, or an interaction between supraspinal and spinal control. However, the outputs generated by our model, consisting only of spinal neurons, suggest that muscle activations can be generated by sensory inputs from loading and hip angles at the spinal level [13].

While there were significant differences in both the inputs (loading and hip angles) and outputs (IEMG) between the different walking speeds and silly walks (Table 1), significant differences were only found in *T*_*2*_ between silly walks and the other walking tests (Figure 6). For normal walking at different speeds, this might imply that an insignificant change in a parameter is sufficient to cause a significant change in the output. Since the control of these parameters, which determine the neuronal properties of the SPG, could come from interneurons, presynaptic inhibition [11], or through descending pathways from supraspinal structures, the insignificant changes might imply that no regulation by the brain or inter-spinal circuitry is required to modulate the activation patterns during walking. Comparing our study to split-belt treadmill locomotion, Morton and Bastian (2006) found that subjects with cerebellar damage were able to perform rapid reactive adjustments to stride length and stance time when their legs were operating at different speeds [14]. Our study corroborates their findings that higher control is not needed to alter the motor output of moving limbs, but the corrections could instead be performed predominantly by spinal structures using available sensory information. It has also been suggested that the same neural circuitry is responsible for gait transitions between walking and running [15]. Thus, while an input from the cerebral cortex is required to initiate a movement, higher command centres need not be recruited to regulate motor output during locomotion regardless of speed.

Grasso et al. [16] suggested that the nervous system attempts to meet motor demands by controlling posture or limb joint motion rather than regulating muscle activations. We agree with their arguments, since we successfully used loading and hip angles as inputs to the SPG model to generate muscle activations. In addition, provided the gait patterns do not result in changes to equilibrium, the same neural network will be utilised [17]. Since the data were captured during steady-state walking, it might also be important that the alternating activations of the flexor and extensor are not disrupted. Perhaps, changes to gait components are secondary, and could result from changes in stride length rather than a different motor control mechanism.

It has been shown that cats with lesions in the motor cortex encountered no problems walking on a flat horizontal surface until they were required to cross obstacles or climb a ladder [18]. In addition, Armstrong and Drew [19] found that pulses measured in the cat’s cortical neurons were unrelated to speed, though muscle activity increased significantly. Therefore, as in humans, no conscious effort is necessary during level walking regardless of walking pace, until an obstacle or a sudden change in the external environment is encountered, when corrective responses are required.

We expected differences in the silly walks as the subjects were intentionally requested to perform a movement unlike normal walking. Since the subjects were consciously aware that they had to perform ‘something silly’, we postulated that the resulting muscle activations were due to a command from the brain. However, we found significant changes only in *T*_*2*_, the adaptation time constant for the TA. Persistent inward currents (PIC) are known to be essential for the firing of motor neurons [20]. It was speculated that PIC are expressed in the extensors from birth, but less so in the flexors [21], because while the extensors are mostly activated during walking, the flexors do not require long-lasting bursts. It would therefore be more economical to modulate the flexors rather than the extensors. Nevertheless, it remains uncertain whether the TA requires more intervention from the brainstem or more neural circuitries than the SOL. The significant difference in *T*_*2*_ could also be due to the SPG model, which requires a strong adaptation effect in generating stable oscillations (Additional file 1) (*T*_*2*, silly walks_ = 0.12 ± 0.17 compared to *T*_*2*, self-selected_ = 0.03 ± 0.04) [10]. The higher *T*_*2*_ value could therefore just be a way for the model to continue generating stable oscillations.

A limitation in this study was the restricted array of silly walks the subjects could perform while walking on a treadmill at a constant speed (an example is shown in ‘Additional file 1’). The movements performed by the subjects still involved an on-going, uninterrupted rhythmic pattern of activation between the antagonistic muscles at the ankle. Since we now know the same neural network is responsible for normal walking at different speeds, future studies can give the subjects a freer choice of the types of silly walks they would like to perform (like those seen in Monty Python’s sketch, *The Ministry of Silly Walks*). In such studies, significant differences in more model parameters might be found.

## Conclusion

We proposed that SPG in the spinal cord can interpret and respond accordingly to velocity-dependent afferent information. Changes in walking speed do not require a different motor control mechanism provided equilibrium is not affected and there is no disruption of the continuous rhythmic patterns produced at the ankle.

## Abbreviations

SPG: Spinal pattern generators; EMG: Electromyographic; SOL: M. soleus; TA: M. tibialis anterior; a: Mutual inhibition (a < 0), self-excitation (a > 0); b: Determine time course of adaptation in neuron; f: Adaptation in neuron; m: Weight of hip angles input; n: Weight of change of hip angles; p: Smoothed force; q: Smoothed hip angles; r: Smoothing coefficient; s: Signal input; T: Determine time course of adaptation in neuron; v: Weight of change of force input; w: Weight of force input; x: Inner state of neuron; y: Neural output; F: Force; HA: Hip angles; Max F: Maximum force; IEMG: Cumulative numerical integration of EMG signals; IEMG_SOL: Cumulative numerical integration of soleus EMG signals; IEMG_TA: Cumulative numerical integration of tibialis anterior EMG signals; MANOVA: Multivariate analysis of variance; ANOVA: Analysis of variance; std: Standard deviation; ave: Average; PIC: Persistent inward currents

## Competing interest

The authors declare that they have no competing interests.

## Authors' contributions

All the authors contributed significantly to the conception and design of the project. SY acquired, analysed and interpreted the data. All authors drafted and revised the manuscript. All authors read and approved the final manuscript.

## Supplementary Material

Additional file 1 One subject performing a silly walk. Click here for file
